# Effects of canagliflozin compared with placebo on major adverse cardiovascular and kidney events in patient groups with different baseline levels of HbA_1c_, disease duration and treatment intensity: results from the CANVAS Program

**DOI:** 10.1007/s00125-021-05524-1

**Published:** 2021-08-26

**Authors:** Tamara K. Young, Jing-Wei Li, Amy Kang, Hiddo J. L. Heerspink, Carinna Hockham, Clare Arnott, Brendon L. Neuen, Sophia Zoungas, Kenneth W. Mahaffey, Vlado Perkovic, Dick de Zeeuw, Greg Fulcher, Bruce Neal, Meg Jardine

**Affiliations:** 1 The George Institute for Global Health, UNSW, Sydney, NSW Australia; 2 The George Institute for Global Health, Imperial College London, London, UK; 3 University of Sydney, Sydney, NSW Australia; 4 Monash University, Melbourne, VIC Australia; 5 Stanford Center for Clinical Research, Department of Medicine, Stanford University School of Medicine, Stanford, CA USA; 6 Department of Clinical Pharmacy and Pharmacology, University Medical Center Groningen, Groningen, the Netherlands

**Keywords:** Baseline HbA_1c_, Complications, Disease duration, Treatment intensity, Type 2 diabetes complexity

## Abstract

**Aims/hypothesis:**

Type 2 diabetes mellitus can manifest over a broad clinical range, although there is no clear consensus on the categorisation of disease complexity. We assessed the effects of canagliflozin, compared with placebo, on cardiovascular and kidney outcomes in the CANagliflozin cardioVascular Assessment Study (CANVAS) Program over a range of type 2 diabetes mellitus complexity, defined separately by baseline intensity of treatment, duration of diabetes and glycaemic control.

**Methods:**

We performed a post hoc analysis of the effects of canagliflozin on major adverse cardiovascular events (MACE) according to baseline glucose-lowering treatments (0 or 1, 2 or 3+ non-insulin glucose-lowering treatments, or insulin-based treatment), duration of diabetes (<10, 10 to 16, >16 years) and HbA_1c_ (≤53.0 mmol/mol [<7.0%], >53.0 to 58.5 mmol/mol [>7.0% to 7.5%], >58.5 to 63.9 mmol/mol [>7.5 to 8.0%], >63.9 to 69.4 mmol/mol [8.0% to 8.5%], >69.4 to 74.9 mmol/mol [>8.5 to 9.0%] or >74.9 mmol/mol [>9.0%]). We analysed additional secondary endpoints for cardiovascular and kidney outcomes, including a combined kidney outcome of sustained 40% decline in eGFR, end-stage kidney disease or death due to kidney disease. We used Cox regression analyses and compared the constancy of HRs across subgroups by fitting an interaction term (*p* value for significance <0.05).

**Results:**

At study initiation, 5095 (50%) CANVAS Program participants were treated with insulin, 2100 (21%) had an HbA_1c_ > 74.9 mmol/mol (9.0%) and the median duration of diabetes was 12.6 years (interquartile interval 8.0–18 years). Canagliflozin reduced MACE (HR 0.86 [95% CI 0.75, 0.97]) with no evidence that the benefit differed between subgroups defined by the number of glucose-lowering treatments, the duration of diabetes or baseline HbA_1c_ (all p-heterogeneity >0.17). Canagliflozin reduced MACE in participants receiving insulin with no evidence that the benefit differed from other participants in the trial (HR 0.85 [95% CI 0.72, 1.00]). Similar results were observed for other cardiovascular outcomes and for the combined kidney outcome (HR for combined kidney outcome 0.60 [95% CI 0.47, 0.77]), with all p-heterogeneity >0.37.

**Conclusions/interpretation:**

In people with type 2 diabetes mellitus at high cardiovascular risk, there was no evidence that cardiovascular and renal protection with canagliflozin differed across subgroups defined by baseline treatment intensity, duration of diabetes or HbA_1c_.

**Graphical abstract:**

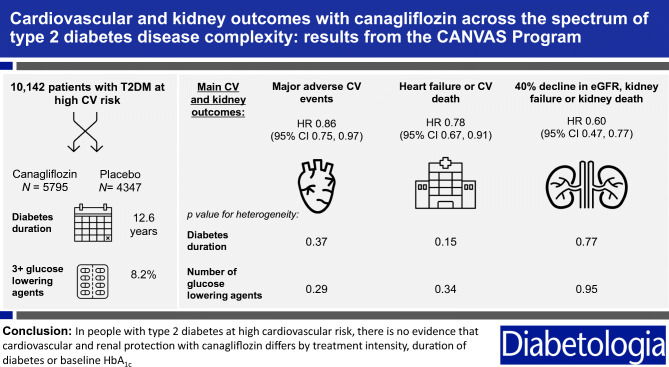

**Supplementary Information:**

The online version of this article (10.1007/s00125-021-05524-1) contains peer-reviewed but unedited supplementary material.

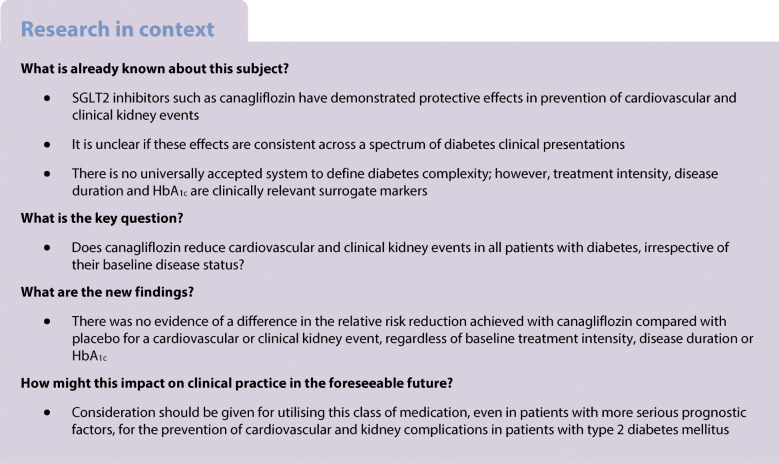



## Introduction

Type 2 diabetes mellitus is a chronic disease, and individuals included in large trials vary widely in terms of their glycaemic control and treatment management strategies. However, there is no universally agreed system for classifying the complexity of diabetes mellitus. A higher level of treatment intensity, increased disease duration and an elevated HbA_1c_ all imply a more complex disease state [1, 2]. The intensity of diabetes treatment is a surrogate marker of diabetes complexity in the context of international guidelines framed around glycaemic control [3]. Disease duration is another possible variable to describe disease complexity and is independently associated with increased morbidity and mortality risk, reflecting an underlying progressive disease course [1, 4, 5]. HbA_1c_ level is a biochemical predictor of the development of microvascular complications [6–8], reflected in HbA_1c_ thresholds appearing as targets for guideline-directed therapy [8–10]. Overall, these variables represent different aspects of a heterogeneous disease.

Sodium–glucose cotransporter 2 (SGLT2) inhibitors reduce cardiovascular events in people with type 2 diabetes mellitus and high cardiovascular risk [11–15]. The SGLT2 inhibitor canagliflozin has also been demonstrated to reduce the progression of chronic kidney disease and prevent clinical kidney events [11, 12]. Despite the requirement of these agents to be filtered at the glomerulus to meet their site of action, canagliflozin shows consistent kidney and cardiovascular protection across declining categories of eGFR [15, 16]. However, the effectiveness of these agents in differing states of glycaemia and diabetes severity is unclear and may plausibly vary depending on the underlying glycaemic control and the extent of pre-existing diabetes-related complications [17–19].

The aim of these analyses was to assess the protective effect of canagliflozin on key cardiovascular and kidney outcomes across a broad range of diabetes severity at treatment initiation. In the absence of a consensus for classification of diabetes severity or complexity, our assessment for the treatment efficacy and safety of canagliflozin in the CANagliflozin cardioVascular Assessment Study (CANVAS) Program on cardiovascular and kidney outcomes was undertaken according to three different variables: diabetes treatment intensity, diabetes duration and baseline HbA_1c_.

## Methods

### Study design and participants

The CANVAS Program comprised two multicentre, double-blind, placebo-controlled, randomised trials (CANVAS and CANagliflozin cardioVascular Assessment Study – Renal [CANVAS-R]). These were similarly conducted, with a prespecified integrated analysis, and with the aim of assessing the cardiovascular efficacy and safety of canagliflozin in participants with type 2 diabetes and either a history of, or at high risk for, CVD. The two trials were scheduled for joint close-out and analysis when at least 688 cardiovascular events had occurred, and the last randomised participant had undergone at least 78 weeks of follow-up [20]. Local institutional ethics committees approved the trial protocols at each site. Trials were registered and details are available online (ClinicalTrials.gov registration no. NCT01032629 and NCT01989754). All participants provided written, informed consent to participate. The trial protocols and statistical analysis plans were published along with the primary CANVAS Program manuscript [11].

### Study population

Entry criteria for both trials included participants with type 2 diabetes (53.0 mmol/mol [7.0%] ≤ HbA_1c_ ≤ 91.3 mmol/mol [10.5%]) who were either ≥30 years old with established atherosclerotic vascular disease or ≥50 years old with two or more cardiovascular risk factors. Risk factors were defined as: duration of diabetes of at least 10 years; systolic blood pressure >140 mmHg while receiving one or more antihypertensive agents; current smoking; microalbuminuria or macroalbuminuria; or high-density lipoprotein cholesterol level of less than 1 mmol/l (38.7 mg/dl).

### Baseline diabetes variable definitions

This post hoc analysis was designed after the main study was published. The analysis plan for the current analyses, including the definition of the baseline subgroups, was prespecified prior to commencement of these analyses.

Participants were divided into subgroups for treatment intensity, disease duration and baseline HbA_1c_, according to the following definitions:

1. **Treatment intensity** Subgroups for treatment intensity were defined by glucose-lowering treatments at baseline study visit and were: zero or one oral glucose-lowering agent, two oral glucose-lowering agents, three or more oral glucose-lowering agents, or any combination of glucose-lowering medication that included insulin therapy.
2. **Disease duration** Subgroups were defined by tertiles of disease duration at baseline, namely: duration of <10 years, 10–16 years or >16 years.
3. **Baseline HbA**_**1c**_ Baseline HbA_1c_ was measured 2 weeks prior to randomisation. Subgroups of HbA_1c_ were defined on clinically accepted categories of HbA_1c_ of ≤53.0 mmol/mol (<7.0%), >53.0 to 58.5 mmol/mol (>7.0% to 7.5%), >58.5 to 63.9 mmol/mol (>7.5% to 8.0%), >63.9 to 69.4 mmol/mol (>8.0% to 8.5%), >69.4 to 74.9 mmol/mol (>8.5% to 9.0%) and >74.9 mmol/mol (>9.0%).

### Randomisation and conduct of the CANVAS Program trials

A web-based response system was used for computer-generated random allocation. In CANVAS, participants were randomly assigned to canagliflozin 100 mg daily, canagliflozin 300 mg daily or placebo. In CANVAS-R, they were randomly assigned to canagliflozin 100 mg daily with potential dose escalation or matching placebo. Face-to-face follow-up was scheduled in three visits during the first year and at 6 month intervals thereafter, with telephone follow-up between face-to-face assessments. Adverse events were collected and reported separately. Ongoing glycaemic management was in accordance with local guidelines. Central endpoint adjudication committees blinded to treatment allocation assessed cardiovascular, kidney and key safety outcomes. The trials were analysed and reported together [11, 20].

### Outcomes

The outcomes selected for this post hoc analysis were the same as those used in the primary reporting of the CANVAS Program [11]. Other prespecified secondary cardiovascular outcomes were death from CVD, myocardial infarction, stroke and a combined outcome comprising either cardiovascular death or admission for heart failure [11].

The kidney outcome was a composite of end-stage kidney disease, kidney death and 40% decrease in eGFR, which was required to be sustained for two consecutive measures of ≥30 days apart or occurring on the last available measure to ensure the measures reflected chronic progression [11]. Albuminuria progression comprised more than a 30% increase in albuminuria and a change either from normoalbuminuria to microalbuminuria or macroalbuminuria, or from microalbuminuria to macroalbuminuria. The Modification of Diet in Renal Disease study equation to define eGFR was used as in the primary analysis. Albuminuria was measured in first morning void urine specimens and calculated as the urinary albumin/creatinine ratio (UACR).

### Statistical analysis

Baseline characteristics with continuous data were described using mean (SD) or, when non-normally distributed, using median (interquartile interval [IQI]). Categorical data were described as frequencies and percentages. Differences in characteristics between subgroups were examined using a linear trend test from generalised linear models for continuous variables and Cochran–Armitage trend test for discrete variables.

HRs for the effect of canagliflozin compared with placebo, 95% CIs and *p* values were estimated with Cox regression models, using an intention-to-treat approach, with stratification according to trial and history of CVD. Comparisons across subgroups were assessed for heterogeneity, with a *p* value of less than 0.05 regarded as significant. The global *p* values for heterogeneity across all subgroups were obtained by fitting an interaction term in the Cox regression model.

Restricted cubic splines using columns that were univariate spline expansions of disease duration and baseline HbA_1c_ were fitted to proportional hazard regression to generate dose–response figures, setting point estimates at the middle of each category, for the major adverse cardiovascular events (MACE) outcome.

All analyses were performed using SAS version 9.4 (SAS Institute, Cary, NC, USA).

## Results

The CANVAS Program included a total of 10,142 participants, 4330 participants in the CANVAS trial and 5812 participants in the CANVAS-R trial. Across the program, 5795 participants were randomised to canagliflozin and 4347 were randomised to placebo. During a mean follow-up of 188.2 weeks, 1011 participants experienced the combined MACE outcome. Canagliflozin reduced MACE (HR 0.86 [95% CI 0.75, 0.97]), and the combined kidney outcome (HR 0.60 [95% CI 0.47, 0.77]), as previously published elsewhere [11].

**Fig. 1:**
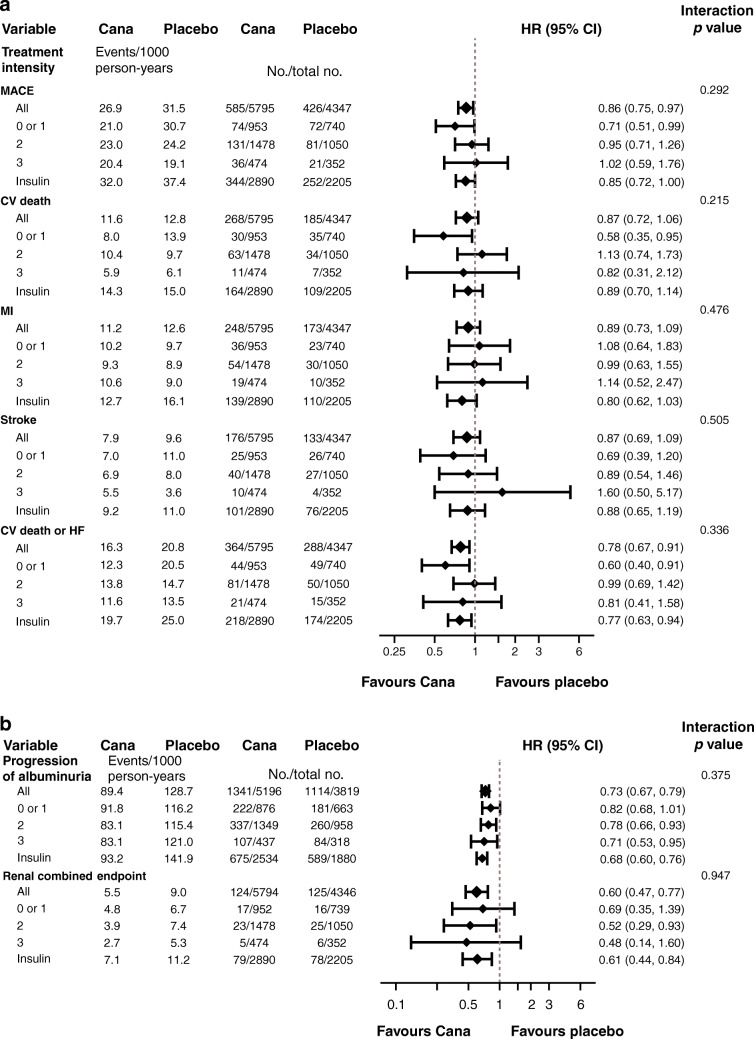
HRs for (**a**) cardiovascular and (**b**) kidney outcomes according to baseline treatment intensity. HRs cannot be directly calculated from event numbers because the trials had different randomisation ratios and different follow-up durations. The follow-up for CANVAS was 295.9 weeks and for CANVAS-R was 108.0 weeks. Cana, canagliflozin; CV, cardiovascular; HF, heart failure; MI, myocardial infarction

**Fig. 2:**
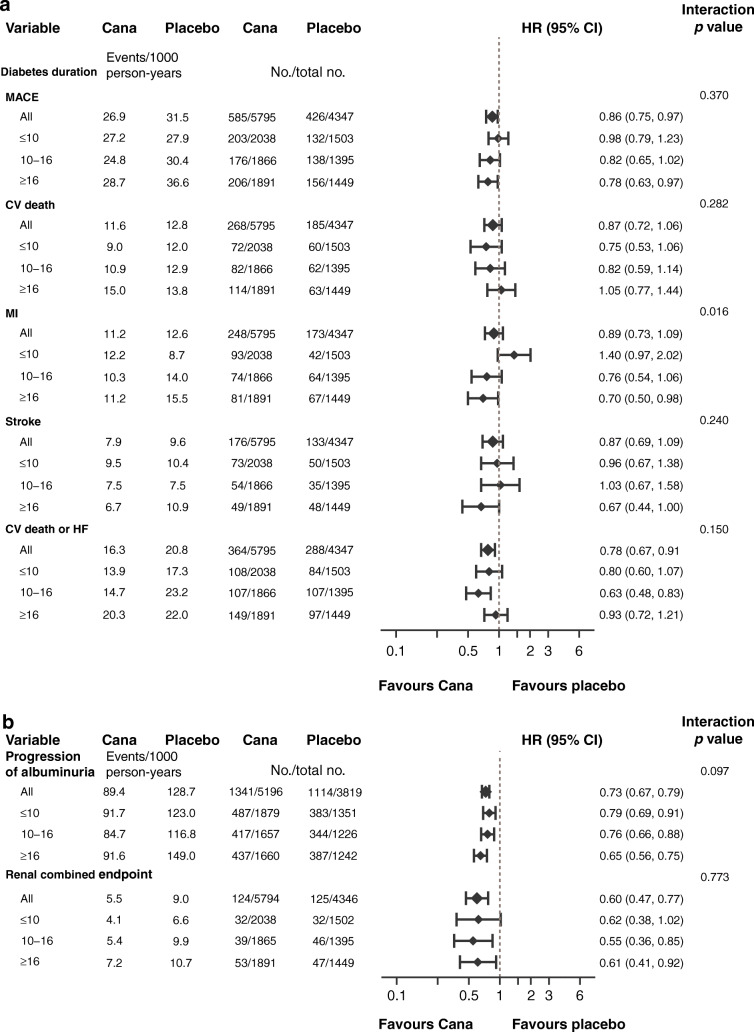
HRs for (**a**) cardiovascular and (**b**) kidney outcomes according to baseline disease duration. HRs cannot be directly calculated from event numbers because the trials had different randomisation ratios and different follow-up durations. The follow-up for CANVAS was 295.9 weeks and for CANVAS-R was 108.0 weeks. CV, cardiovascular; HF, heart failure; MI, myocardial infarction

At study initiation, 5095 (50%) CANVAS Program participants were treated with insulin. The median duration of diabetes was 12.6 years (interquartile interval 8.0–18 years). In total, 2100 participants (21%) had a baseline HbA_1c_ greater than 74.9 mmol/mol (9.0%). In general, the markers of disease severity at baseline were consistent between the three variables (Table 1). Mean HbA_1c_ was lowest in the participants on zero or one agent at baseline (mean 64 mmol/mol [8.0%, SD 0.9%]) and highest in those on insulin therapy (mean 68 mmol/mol [8.4%, SD 0.9%]). Mean disease duration was shortest in the participants on zero or one agent at baseline (mean 8.5 years, SD 6.2 years) and longest in those on insulin therapy (mean 16.3 years, SD 7.7 years). Mean disease duration was shortest for those with the lowest HbA_1c_ category at baseline (mean 11.0 years, IQI 6–16 years) and longest for those with the highest three HbA_1c_ categories at baseline (all mean 13.0 years) (Electronic supplementary material [ESM] Figs. 1, 2; ESM Tables 1–3).

**Table 1:** Baseline characteristics according to subgroups of each variable of diabetes complexity

Characteristic	Intensity of treatment					Disease duration (years)				Baseline HbA_1c_ (mmol/mol [%])						
	0 or 1 agents	2 agents	3+ agents	Insulin	*p*	≤10	10–16	≥16	*p*	53 (<7.0)	53.0–58.5 (7.0–7.5)	58.5–63.9 (7.5–8.0)	63.9–69.4 (8.0–8.5)	69.4–74.9 (8.5–9.0)	>74.9 (>9.0)	*p*
Number	1693	2528	826	5095		3541	3261	3340		746	1940	2147	1793	1416	2100	
Mean age (years)	63.4	62.9	62.9	63.5	0.723	61.2	62.9	65.9	<0.001	64.0	64.5	64.1	63.2	62.7	61.7	<0.001
Female (%)	36.4	37.1	28.2	36.2	0.535	33.9	36.7	37.0	0.006	32.4	32.2	35.9	36.7	35.8	39.5	<0.001
Heart failure (%)	18.2	16.2	5.0	13.8	<0.001	18.7	13.2	11.1	<0.001	12.9	12.7	13.2	13.6	15.1	18.0	<0.001
Mean duration of diabetes (years)	8.5	11.3	13.9	16.3	<0.001	6.0	12.8	22.3	<0.001	11.0	12.0	12.3	13.0	13.0	13.0	<0.001
CVD (%)	69.5	64.0	54.0	67.0	0.732	79.6	51.1	65.1	<0.001	67.7	67.1	65.6	63.3	66.1	65.2	0.143
Mean BMI (kg/m^2^)	31.4	30.9	31.2	32.8	<0.001	32.0	32.2	31.7	0.074	31.9	31.7	31.8	31.8	32.2	32.3	0.02
Mean HbA_1c_ (mmol/mol)	64	66	66	68	<0.001	66	67	67	<0.001	51	56	62	67	73	83	<0.001
Mean HbA_1c_ (%)	8.0	8.2	8.2	8.4	<0.001	8.2	8.3	8.3	<0.001	6.8	7.3	7.8	8.3	8.8	9.7	<0.001

Albuminuria was different among HbA_1c_ subgroups (*p* < 0.05), although there was no discernible difference in eGFR. For example, participants in the lowest HbA_1c_ subgroup had less albuminuria (median UACR 1.1 mg/mmol, IQI 1.0–2.6 mg/mmol) compared with those in the highest HbA_1c_ category (median UACR 2.0 mg/mmol, IQI 0.9–8.0 mg/mmol). Both albuminuria and eGFR were different between disease duration subgroups (*p* < 0.05 for both). Participants with the shortest disease duration had higher eGFR and lower albuminuria than those with the longest disease duration (mean eGFR 80.0 ml min^−1^ [1.73 m]^−2^, SD 20.3 ml min^−1^ [1.73 m]^−2^, median UACR 1.2 mg/mmol, IQI 0.7–3.4 mg/mmol; and mean eGFR 71.6 ml min^−1^ [1.73 m]^−2^, SD 19.9 ml min^−1^ [1.73 m]^−2^, median UACR 1.8 mg/mmol, IQI 0.8–7.3 mg/mmol, respectively). Similarly, both albuminuria and eGFR were different in treatment intensity subgroups (*p* < 0.05 for both). Participants with the lowest intensity of treatment had higher eGFR and lower albuminuria than those receiving insulin-based treatment (mean eGFR 77.7 ml min^−1^ [1.73 m]^−2^, SD 20.9 ml min^−1^ [1.73 m]^−2^, median UACR 1.1 mg/mmol, IQI 0.7–2.8 mg/mmol; and mean eGFR 74.1 ml min^−1^ [1.73 m]^−2^, SD 20.6 ml min^−1^ [1.73 m]^−2^, median UACR 1.7 mg/mmol, IQR 0.8–7.0 mg/mmol, respectively) (ESM Tables 1–3).

### Outcomes according to treatment intensity

When considering the reduction in MACE for the overall trial population (HR 0.86 [CI 0.75, 0.97]), there was no evidence that the benefit differed across subgroups of baseline treatment intensity (HR 0.71 [CI 0.51, 0.99], HR 0.95 [CI 0.71, 1.26], HR 1.02 [CI 0.59, 1.76], HR 0.85 [CI 0.72, 1.0] for 0 or 1, 2 or 3+ glucose-lowering agents, or insulin-based therapy, respectively; p-heterogeneity 0.292). The point estimates of the HRs suggested a potential benefit for the individual cardiovascular outcomes; however, the CIs around the overall effect and the effects within each subgroup were wide and overlapped the null, so it was not possible to draw definitive conclusions (Fig. 1a).

There was consistency across treatment intensity categories for the reduction of the kidney disease outcome of sustained 40% decrease in eGFR, end-stage kidney disease or kidney death, and for the protection against progression of albuminuria (p-heterogeneity 0.375 and 0.947, respectively) (Fig. 1b).

### Outcomes according to disease duration

**Fig. 3:**
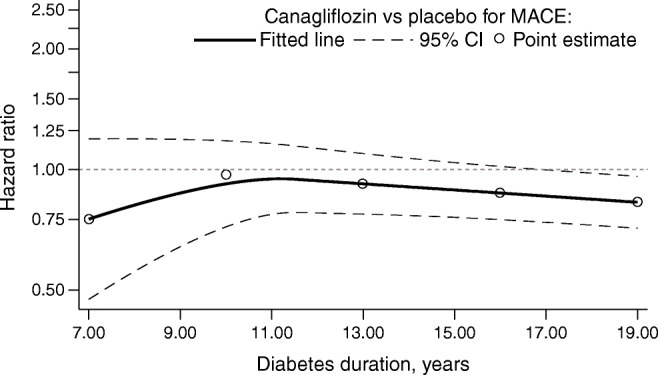
Cubic spline model for HR for MACE with canagliflozin vs placebo according to baseline disease duration. The *y*-axis is plotted on a log scale

The reduction in MACE was similar across subgroups defined by duration of diabetes (*p* = 0.37). The point estimates of the HRs suggested a potential benefit for the individual cardiovascular outcomes; however, once again, the CIs around the overall effect and the effects within each subgroup were wide and overlapped the null, so it was not possible to draw definitive conclusions (Fig. 2a). The effect of canagliflozin on myocardial infarction differed across subgroups, with no benefit seen for myocardial infarction in those with disease duration of less than 10 years (*p* for heterogeneity between defined subgroups = 0.01). However, when considering the relative reduction in MACE, there was no evidence that the benefit differed across the range of disease duration when tested as a continuous variable (Fig. 3).

There was no evidence that the benefit differed for a reduction of the kidney disease outcome and prevention of the progression of albuminuria across subgroups of disease duration (*p* = 0.773 and 0.097, respectively) (Fig. 2b).

### Outcomes according to baseline HbA_1c_

**Fig. 4:**
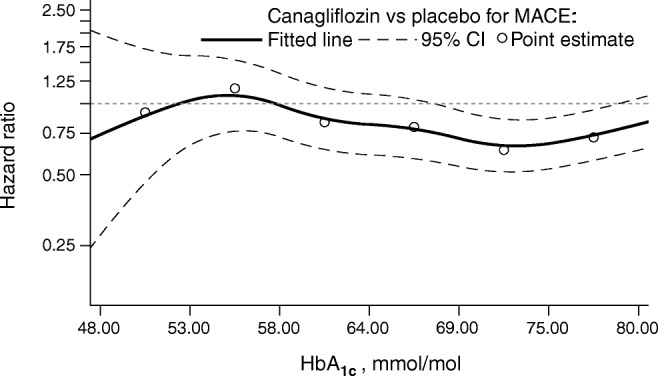
Cubic spline model for HR for MACE with canagliflozin vs placebo according to baseline HbA_1c_. The *y*-axis is plotted on a log scale

**Fig. 5:**
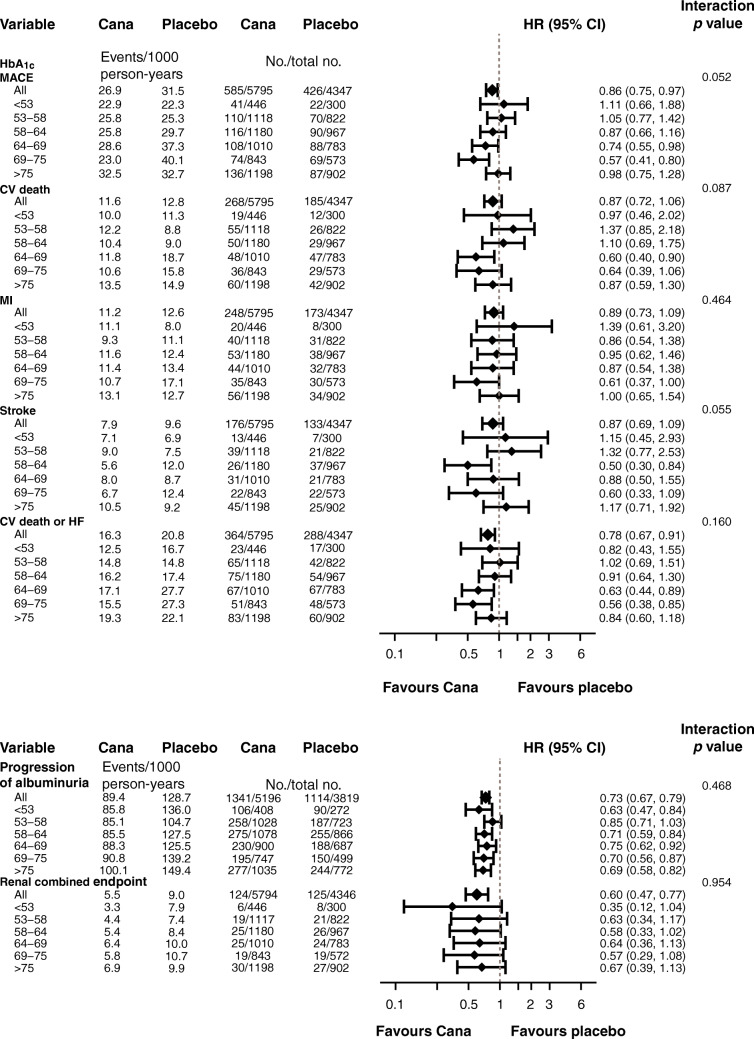
HRs for (**a**) cardiovascular and (**b**) kidney outcomes according to baseline HbA_1c_. HRs cannot be directly calculated from event numbers because the trials had different randomisation ratios and different follow-up durations. The follow-up for CANVAS was 295.9 weeks and for CANVAS-R was 108.0 weeks. CV, cardiovascular; HF, heart failure; MI, myocardial infarction

For the outcome of MACE, numerically, the effects of canagliflozin appeared to be greater in the HbA_1c_ subgroups between 53.0 and 74.9 mmol/mol (7.0% and 9.0%), although this association was not seen for other cardiovascular outcomes or in analyses of HbA_1c_ as a continuous variable (Fig. 4). The point estimates of the HRs suggested a potential benefit for the individual cardiovascular outcomes; however, with the CIs around the overall effect and the effects within each subgroup overlapping the null, it was not possible to draw definitive conclusions (Fig. 5a).

There was no evidence that the benefit of canagliflozin for a reduction in the kidney disease outcome, and protection against progression of albuminuria, differed between subgroups defined by baseline HbA_1c_ (*p* = 0.954 and 0.468, respectively) (Fig. 5b).

## Discussion

In this secondary post hoc analysis of a large, multicentre randomised controlled trial, participants with type 2 diabetes randomised to canagliflozin received similar cardioprotective and renoprotective effects regardless of their baseline HbA_1c_, disease duration or intensity of diabetes treatment. Canagliflozin was associated with a reduction in cardiovascular death and stroke, even in participants in the highest disease duration tertile, representing at least 16 years with the disease. Canagliflozin had consistent effects for MACE, including in participants receiving insulin at baseline. This implies a possible drug effect on the prevention of macrovascular complications that is not attenuated in those with a more complex disease state. In patients randomised to receive canagliflozin, kidney protection was observed across all variables of disease severity, including maximum treatment intensity, long disease duration and higher baseline HbA_1c_. Regardless of how it is defined, a higher level of baseline disease complexity or severity does not appear to pose a threshold for the cardiovascular and kidney benefits of canagliflozin.

In the absence of a clear consensus on diabetes severity categorisation, an analysis of outcomes using these three plausible variables in a trial population with a range of underlying disease complexity was undertaken. There is no universally agreed system for classifying disease severity or complexity in major international guidelines that is the equivalent of the Kidney Disease: Improving Global Outcomes (KDIGO) CKD stage classification system [21, 22]. The observed concordance in findings across these three potential variables of diabetes disease complexity suggests a possible approach to systematically evaluating a disease with broad clinical presentation. This multifaceted approach for describing diabetes complexity may provide a framework for future analyses to capture a wide spectrum of disease. Overall, our broad method of describing baseline diabetes complexity, and the reproducibility of findings across all three variables, increases the generalisability of these findings to a wide range of people with type 2 diabetes.

Analyses of trial efficacy according to variables of disease complexity have been incompletely reported [14, 23, 24]. The Dapagliflozin Effect on Cardiovascular Events–Thrombolysis in Myocardial Infarction 58 (DECLARE-TIMI58) trial demonstrated heterogeneous MACE outcomes and attenuated renoprotective outcomes in patients with type 2 diabetes disease duration greater than 20 years who were randomised to receive an SGLT2 inhibitor [25]. Additionally, while major trials for cardiovascular outcomes in SGLT2 inhibitors all consider baseline HbA_1c_, albeit with a range of entry criteria, only one has reported outcomes according to baseline HbA_1c_, demonstrating that the cardioprotective effects of empagliflozin were independent of baseline glycaemic control [26]. This is concordant with our findings. Other trials do not routinely analyse outcomes according to the number of agents that patients are prescribed at study entry, or the proportion of individual agents that are used [27]. The CANVAS Program had a higher proportion of insulin use at baseline compared with other major trials, suggesting a higher proportion of participants with more complex type 2 diabetes mellitus, yet still reported favourable outcomes in this subgroup of participants. Other recent trials examining cardiovascular and kidney outcomes for novel glucose-lowering agents for diabetes other than SGLT2 inhibitors, while including a broad range of participants, also do not routinely attempt to stratify participants for baseline variables of pre-existing treatment intensity or disease duration [28–30]. Additionally, in trials for another novel glucose-lowering agent, glucagon-like peptide-1 (GLP-1) receptor agonists, the reported cardiovascular and kidney benefits are evident across a range of baseline diabetes variables, including those with longstanding disease and higher baseline HbA_1c_. While individually reported in some trials, our approach represents a novel approach to examine these three variables in parallel.

The three variables used in this analysis have clinical utility as an acceptable marker for disease complexity. There is no standard algorithm or risk score for defining this. Each of them is associated with the development of microvascular and macrovascular complications in patients with type 2 diabetes mellitus. Many patients with type 2 diabetes mellitus have an increase in treatment escalation in order to achieve glycaemic targets over time, ultimately with a high proportion progressing to insulin-based therapy [4, 31]. Additionally, other general definitions of chronic disease severity or complexity incorporate the concept of disease burden, as defined by treatment requirements such as insulin or increasing number of medications [2, 32]. The duration of type 2 diabetes mellitus is an independent, continuous risk factor for both CVD and chronic kidney disease [5, 33]. Longer duration of disease is also associated with an increased risk of mortality, and therefore is representative of a more severe disease state [34–36]. Finally, an elevated HbA_1c_ is continuously associated with both the risk of complications and mortality [8, 37].

Poor glycaemic control is an established risk factor for increased macrovascular complications and higher rates of cardiovascular mortality [8, 38]. This analysis did not detect a significant difference in treatment effect on the outcome of MACE across subgroups according to baseline HbA_1c_ (*p* = 0.052, although there was a numeric trend to a greater benefit in those with higher HbA_1c_ at baseline). These results should be regarded cautiously given the multiple comparisons made and the post hoc nature of the analyses.

There were some risk factors that were higher in young populations. The CANVAS Program inclusion criteria were devised to recruit participants at high risk of CVD [20]. Younger participants (30–49 years) were required to have a history of symptomatic vascular disease while the presence of two or more risk factors was sufficient in participants aged 50 years and over. By their nature, the inclusion criteria introduce selection bias. This post hoc analysis of a randomised controlled trial was not designed to assess epidemiological factors.

The clinical implications of this analysis relate to both treatment effects and underlying baseline risk profile of patients with type 2 diabetes mellitus. In this analysis, kidney protection was observed in all variables of disease complexity. Our findings in patient groups with a higher risk profile of diabetic kidney disease, such as longer disease duration or elevated baseline HbA_1c_, are encouraging in this population group who are inherently at risk of disease progression, and concordant with the other recent large-scale trial data specifically designed to test this hypothesis [12].

The strengths of this study are the multicentre, randomised controlled trial design conducted at a high standard and with a very large number of participants. The cardiovascular and kidney outcomes were prespecified and adjudicated by expert committees.

This secondary analysis also has inherent limitations applicable to any post hoc analysis of a randomised trial. The CANVAS study was not designed to test these subgroup analyses, which should be regarded as exploratory, nor were there adjustments for multiple comparisons. Further, relatively small numbers of participants with eGFR <45 ml min^−1^ [1.73 m]^−2^ were recruited, which limits our ability to draw definitive conclusions about the effects of canagliflozin in participants with significantly reduced kidney function. The CANVAS study recruited participants with diabetes and at high cardiovascular risk; therefore, the results may not generalise to other populations. However, the consistency of effect size on a range of cardiovascular and kidney outcomes supports the likely beneficial effects of SGLT2 inhibitors in patients with type 2 diabetes mellitus at high cardiovascular risk.

### Conclusions

Canagliflozin has beneficial cardiovascular and kidney outcomes that are evident across a wide range of diabetes control and levels of complexity in patient groups who have an underlying elevated risk of CVD. Consideration for the clinical utility of SGLT2 inhibitors in the prevention of cardiovascular and kidney complications of diabetes across a spectrum of disease, even in those patient groups with more serious prognostic factors, is warranted.

## Supplementary Information

ESM (PDF 181 kb)

## Data Availability

Data from the CANVAS Program will be made available in the public domain via the Yale University Open Data Access Project (http://yoda.yale.edu/) once the product and relevant indications studied have been approved by regulators in Europe and the United States and the study has been completed for 18 months. Data are available from the authors upon reasonable request.

## Abbreviations

CANVAS: CANagliflozin cardioVascular Assessment Study
CANVAS-R: CANagliflozin cardioVascular Assessment Study – Renal
IQI: Interquartile interval
MACE: Major adverse cardiovascular events
SGLT2: Sodium–glucose cotransporter 2
UACR: Urinary albumin/creatinine ratio
