# Supplementary material for: Effects of canagliflozin compared with placebo on major adverse cardiovascular and kidney events in patient groups with different baseline levels of HbA1c, disease duration and treatment intensity: results from the CANVAS Program
Source: Diabetologia. 2021 Aug 26;64(11):2402–14. doi: 10.1007/s00125-021-05524-1 (PMC8494676; doi:10.1007/s00125-021-05524-1)

Supplementary Tables and Figures

ESM Table 1: Baseline characteristics according to treatment intensity

ESM Table 2: Baseline characteristics according to diabetes duration

ESM Table 3: Baseline characteristics according to baseline HbA_{1c}

ESM Fig. 1: Histogram of disease duration

ESM Fig. 2: Histogram of baseline HbA_{1c}

ESM Table 1 Baseline characteristics according to treatment intensity

**Characteristic**	**0 or 1 (n=1693)**	**2 (n=2528)**	**3+ (n=826)**	**Insulin (n=5095)**	**p**
Age — years	63.4±8.8	62.9±8.5	62.9±7.8	63.5±8.0	0.723
Female sex — no. (%)	617 (36.4%)	939 (37.1%)	233 (28.2%)	1844 (36.2%)	0.535
Race — no. (%)					<0.001
White	1351 (79.8%)	1927 (76.2%)	567 (68.6%)	4099 (80.5%)	
Asian	175 (10.3%)	341 (13.5%)	203 (24.6%)	565 (11.1%)	
Black	60 (3.5%)	91 (3.6%)	21 (2.5%)	164 (3.2%)	
Other	107 (6.4%)	169 (6.7%)	35 (4.3%)	267 (5.2%)	
Current smoker — no. (%)	386 (22.8%)	468 (18.5%)	150 (18.2%)	802 (15.7%)	<0.001
History of hypertension — no. (%)	1501 (88.7%)	2249 (89.0%)	734 (88.9%)	4641 (91.1%)	<0.001
History of heart failure — no. (%)	308 (18.2%)	409 (16.2%)	41 (5.0%)	703 (13.8%)	<0.001
Duration of diabetes — year	8.5±6.2	11.3±6.6	13.9±6.6	16.3±7.7	<0.001
History of atherosclerotic vascular disease — no. (%)					
Coronary	1014 (59.9%)	1354 (53.6%)	387 (46.9%)	2966 (58.2%)	0.439
Cerebrovascular	321 (19.0%)	515 (20.4%)	129 (15.6%)	993 (19.5%)	0.901
Peripheral	303 (17.9%)	505 (20.0%)	91 (11.0%)	1214 (23.8%)	<0.001
History of cardiovascular disease — no. (%)	1177 (69.5%)	1618 (64.0%)	446 (54.0%)	3415 (67.0%)	0.732
History of amputation — no. (%)	13 (0.8%)	28 (1.1%)	8 (1.0%)	189 (3.7%)	<0.001

Body-mass index	31.4±5.9	30.9±5.5	31.2±6.0	32.8±6.0	<0.001
Blood pressure — mm Hg					
Systolic	136.0±14.9	135.6±15.2	135.7±15.5	137.5±16.3	0.002
Diastolic	79.4±9.3	78.5±9.1	77.5±9.6	76.8±9.9	<0.001
Glycated hemoglobin —mmol/mol(*%*)	64 (*8.0±0.9*)	67 (*8.2±0.9*)	66 (*8.2±0.9*)	68 (*8.4±0.9*)	<0.001
Cholesterol — mmol/liter					
Total	4.6±1.2	4.5±1.2	4.1±1.0	4.3±1.1	<0.001
HDL	1.2±0.3	1.2±0.3	1.2±0.3	1.2±0.3	0.574
LDL	2.5±1.0	2.4±1.0	2.1±0.8	2.2±0.9	<0.001
Ratio of LDL to HDL	2.2±1.0	2.1±1.0	1.9±0.9	2.0±0.9	<0.001
Triglycerides — mmol/liter	2.1±1.4	2.1±1.3	1.9±1.3	2.0±1.5	<0.001
eGFR — ml/min/1.73 m^2	77.7±20.9	79.7±19.6	78.9±19.9	74.1±20.6	<0.001
Albumin measurements					<0.001
Median albumin-to-creatinine ratio (interquartile range)	10.1 (6.1, 25.1)	11.3 (6.4, 31.6)	11.0 (6.2, 34.6)	14.6 (7.2, 62.3)	<0.001
< 30	1294 (77.6%)	1852 (74.1%)	594 (72.4%)	3267 (64.8%)	<0.001
30 – 300	297 (17.8%)	525 (21.0%)	180 (22.0%)	1264 (25.1%)	
> 300 - <= 3000	68 (4.1%)	120 (4.8%)	45 (5.5%)	470 (9.3%)	
> 3000	8 (0.5%)	4 (0.2%)	1 (0.1%)	44 (0.9%)	

ESM Table 2: Baseline characteristics according to diabetes duration

**Characteristic**	Dur<=10 yr (n=3541)	Dur 10~16 yr (n=3261)	Dur >=16 yr (n=3340)	p
Age — years	61.2±8.6	62.9±7.7	65.9±7.6	<0.001
Female sex — no. (%)	1200 (33.9%)	1196 (36.7%)	1237 (37.0%)	0.006
Race — no. (%)				0.484
White	2755 (77.8%)	2575 (79.0%)	2614 (78.3%)	
Asian	461 (13.0%)	399 (12.2%)	424 (12.7%)	
Black	131 (3.7%)	107 (3.3%)	98 (2.9%)	
Other	189 (5.5%)	180 (5.5%)	204 (6.1%)	
Current smoker — no. (%)	769 (21.7%)	613 (18.8%)	424 (12.7%)	<0.001
History of hypertension — no. (%)	3134 (88.5%)	2943 (90.3%)	3048 (91.3%)	<0.001
History of heart failure — no. (%)	661 (18.7%)	429 (13.2%)	371 (11.1%)	<0.001
Duration of diabetes — yr	6.0±2.9	12.8±1.6	22.3±5.9	<0.001
History of atherosclerotic vascular disease — no. (%)				
Coronary	2302 (65.0%)	1512 (46.4%)	1907 (57.1%)	<0.001
Cerebrovascular	781 (22.1%)	538 (16.5%)	639 (19.1%)	0.002
Peripheral	751 (21.2%)	569 (17.4%)	793 (23.7%)	<0.001
History of cardiovascular disease — no. (%)	2817 (79.6%)	1666 (51.1%)	2173 (65.1%)	<0.001
History of amputation — no. (%)	43 (1.2%)	71 (2.2%)	124 (3.7%)	<0.001
Body-mass index‖	32.0±6.0	32.2±5.9	31.7±5.9	0.074

Blood pressure — mm Hg				
Systolic	134.9±15.3	137.4±15.3	137.8±16.5	<0.001
Diastolic	79.2±9.3	78.5±9.5	75.3±9.7	<0.001
Glycated hemoglobin —mmol/mol (*%)*	66 (*8.2±1.0)*	67 (*8.3±0.9*)	67 (*8.3±0.9*)	<0.001
Cholesterol — mmol/liter				
Total	4.5±1.2	4.4±1.1	4.2±1.1	<0.001
HDL	1.1±0.3	1.2±0.3	1.2±0.3	<0.001
LDL	2.4±1.0	2.3±0.9	2.2±0.9	<0.001
Ratio of LDL to HDL	2.2±1.0	2.0±0.9	1.9±0.9	<0.001
Triglycerides — mmol/liter	2.2±1.7	2.0±1.3	1.8±1.2	<0.001
eGFR — ml/min/1.73 m^2	80.0±20.3	77.5±20.5	71.6±19.9	<0.001
Albumin measurements				
Median albumin-to-creatinine ratio (interquartile range)	10.7 (6.2, 29.9)	12.2 (6.6, 42.0)	15.6 (7.4, 64.4)	<0.001
< 30	2625 (75.0%)	2267 (70.2%)	2115 (64.0%)	<0.001
30 – 300	699 (20.0%)	697 (21.6%)	870 (26.3%)	
> 300 - <= 3000	161 (4.6%)	249 (7.7%)	293 (8.9%)	
> 3000	14 (0.4%)	15 (0.5%)	28 (0.9%)	
Drug type				<0.001
0 or 1	1096 (31.0%)	408 (12.5%)	189 (5.7%)	
2	1163 (32.8%)	838 (25.7%)	527 (15.8%)	
3	231 (6.5%)	337 (10.3%)	258 (7.7%)	
Insulin	1051 (29.7%)	1678 (51.5%)	2366 (70.8%)	

ESM Table 3: Baseline characteristics according to baseline HbA1c

**Characteristic**							**p**
HbA1c mmol/mol	**53**	**53.0-58.5**	**58.5-63.9**	**63.9-69.4**	**69.4-74.9**	**>75.0**	
%	*<7* (n=746)	*7-7.5* (n=1940)	*7.5-8* (n=2147)	*8-8.5* (n=1793)	*8.5-9* (n=1416)	*>9* (n=2100)	
Age — yr	64.0 (8.0)	64.5 (8.2)	64.1 (8.2)	63.2 (8.2)	62.7 (8.1)	61.7 (8.2)	<0.001
Female sex — no. (%)	242 (32.4%)	625 (32.2%)	771 (35.9%)	658 (36.7%)	507 (35.8%)	830 (39.5%)	<0.001
Race — no. (%)							<0.001
White	594 (79.6%)	1569 (80.9%)	1731 (80.6%)	1396 (77.9%)	1087 (76.8%)	1567 (74.6%)	
Asian	80 (10.7%)	234 (12.1%)	219 (10.2%)	230 (12.8%)	211 (14.9%)	310 (14.8%)	
Black	23 (3.1%)	46 (2.4%)	59 (2.7%)	57 (3.2%)	45 (3.2%)	106 (5.0%)	
Other	49 (6.6%)	91 (4.7%)	138 (6.4%)	110 (6.1%)	73 (5.2%)	117 (5.6%)	
Current smoker — no. (%)	147 (19.7%)	324 (16.7%)	359 (16.7%)	309 (17.2%)	254 (17.9%)	413 (19.7%)	0.090
History of hypertension — no. (%)	685 (91.8%)	1734 (89.4%)	1934 (90.1%)	1609 (89.7%)	1277 (90.2%)	1886 (89.8%)	0.609
History of heart failure — no. (%)	96 (12.9%)	246 (12.7%)	284 (13.2%)	244 (13.6%)	214 (15.1%)	377 (18.0%)	<0.001
Duration of diabetes — years	11.0 (6.0, 16.0)	12.0 (7.0, 17.0)	12.3 (8.0, 18.0)	13.0 (9.0, 18.0)	13.0 (9.2, 19.0)	13.0 (9.0, 18.0)	<0.001

History of atherosclerotic vascular disease — no. (%)							
Coronary	426 (57.1%)	1144 (59.0%)	1202 (56.0%)	972 (54.2%)	808 (57.1%)	1169 (55.7%)	0.108
Cerebrovascular	149 (20.0%)	373 (19.2%)	427 (19.9%)	331 (18.5%)	280 (19.8%)	398 (19.0%)	0.607
Peripheral	146 (19.6%)	367 (18.9%)	437 (20.4%)	380 (21.2%)	315 (22.2%)	468 (22.3%)	0.003
History of cardiovascular disease — no. (%)	505 (67.7%)	1301 (67.1%)	1409 (65.6%)	1135 (63.3%)	936 (66.1%)	1370 (65.2%)	0.143
History of amputation — no. (%)	11 (1.5%)	51 (2.6%)	40 (1.9%)	51 (2.8%)	33 (2.3%)	52 (2.5%)	0.320
Body-mass index	31.9 (5.9)	31.7 (5.9)	31.8 (5.9)	31.8 (5.8)	32.2 (6.0)	32.3 (6.1)	0.020
Blood pressure — mm Hg							
Systolic	135.3 (15.7)	136.4 (15.5)	136.6 (15.5)	136.8 (15.7)	137.6 (15.8)	136.6 (16.3)	0.006
Diastolic	76.6 (9.8)	77.1 (9.3)	77.4 (9.6)	77.8 (9.6)	78.3 (10.1)	78.5 (9.7)	<0.001
Glycated hemoglobin — mmol/mol	51	56	62	67	73	9.7 (0.5)	<0.001
%	*6.8* (0.2)	*7.3 (0.1)*	*7.8 (0.1)*	*8.3 (0.1)*	*8.8 (0.1)*		
Cholesterol — mmol/liter							
Total	4.2 (1.1)	4.3 (1.1)	4.3 (1.2)	4.4 (1.2)	4.4 (1.1)	4.5 (1.2)	<0.001
HDL	1.2 (0.3)	1.2 (0.3)	1.2 (0.3)	1.2 (0.3)	1.2 (0.3)	1.2 (0.3)	<0.001
LDL	2.2 (0.9)	2.2 (0.9)	2.3 (0.9)	2.3 (0.9)	2.4 (0.9)	2.4 (1.0)	<0.001
Ratio of LDL to HDL	1.9 (0.9)	2.0 (0.9)	2.0 (0.9)	2.1 (0.9)	2.1 (0.9)	2.1 (1.0)	<0.001

Triglycerides — mmol/liter	1.5 (1.1, 2.0)	1.6 (1.2, 2.3)	1.7 (1.2, 2.3)	1.7 (1.2, 2.3)	1.7 (1.3, 2.5)	1.8 (1.3, 2.7)	<0.001
eGFR — ml/min/1.73 m^2	75.9 (19.6)	75.4 (19.5)	76.6 (19.8)	76.0 (21.0)	77.0 (20.8)	77.5 (21.8)	0.011
Albumin measurements							<0.001
Median albumin-to-creatinine ratio (interquartile range)	9.3 (5.9, 22.9)	10.2 (6.0, 28.4)	11.2 (6.4, 35.3)	12.1 (6.8, 42.5)	15.1 (7.4, 59.4)	17.6 (7.7, 70.6)	<0.001
< 30	587 (78.7%)	1444 (74.4%)	1547 (72.1%)	1234 (68.8%)	916 (64.7%)	1279 (60.9%)	<0.001
30 – 300	115 (15.4%)	352 (18.1%)	445 (20.7%)	401 (22.4%)	365 (25.8%)	588 (28.0%)	
> 300 - <= 3000	25 (3.4%)	109 (5.6%)	124 (5.8%)	131 (7.3%)	115 (8.1%)	199 (9.5%)	
> 3000	5 (0.7%)	8 (0.4%)	10 (0.5%)	10 (0.6%)	9 (0.6%)	15 (0.7%)	

ESM Fig. 1: Histogram of disease duration

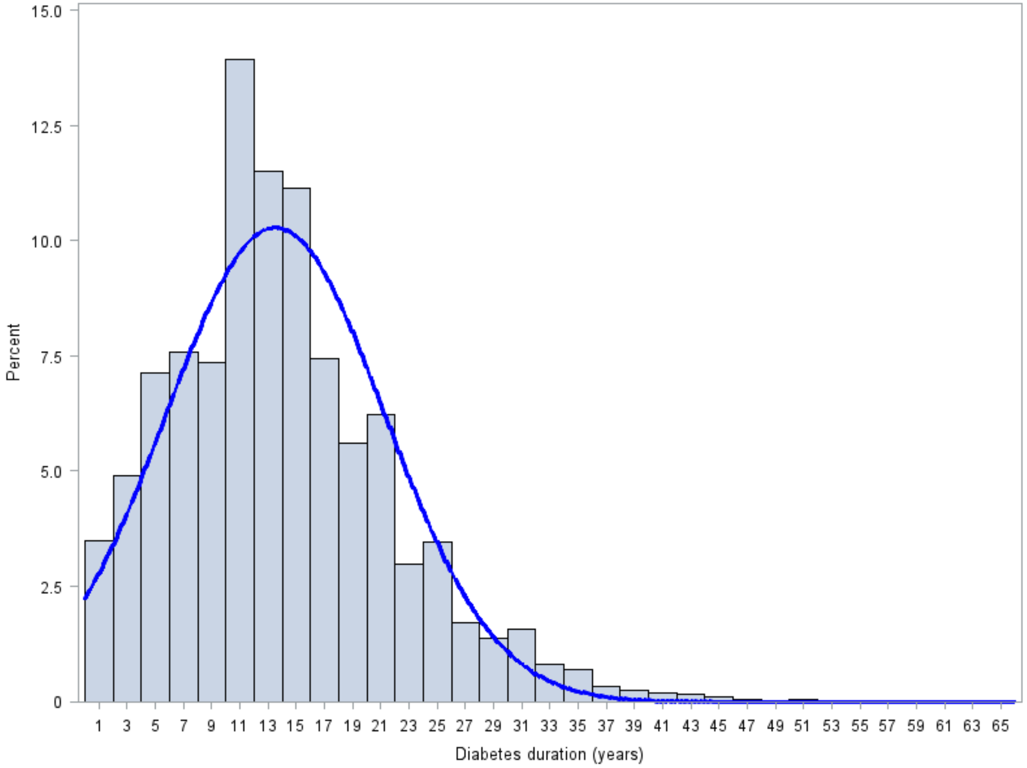

ESM Fig. 2: Histogram of baseline HbA_{1c}

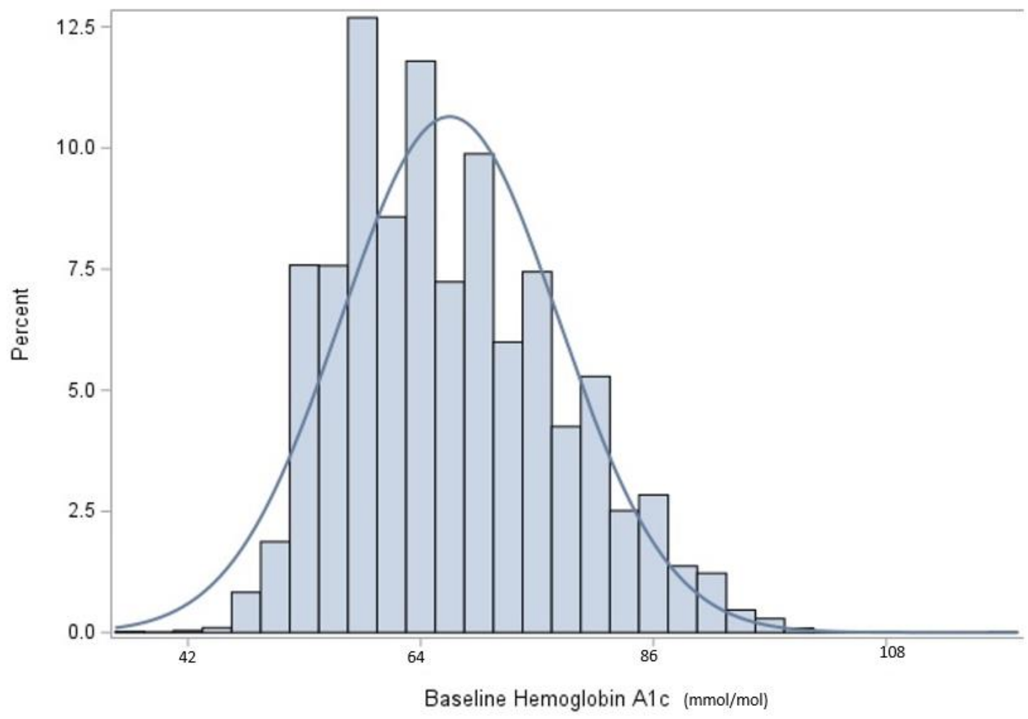

Supplement: Supplementary file 1 — (PDF 181 kb) [file 125_2021_5524_MOESM1_ESM.pdf]
